# Retraction of Activation of Rac1 by Src-dependent phosphorylation of Dock180^Y1811^ mediates PDGFRα-stimulated glioma tumorigenesis in mice and humans

**DOI:** 10.1172/JCI198611

**Published:** 2025-09-16

**Authors:** Haizhong Feng, Bo Hu, Kun-Wei Liu, Yanxin Li, Xinghua Lu, Tao Cheng, Jia-Jean Yiin, Songjian Lu, Susan Keezer, Tim Fenton, Frank B. Furnari, Ronald L. Hamilton, Kristiina Vuori, Jann N. Sarkaria, Motoo Nagane, Ryo Nishikawa, Webster K. Cavenee, Shi-Yuan Cheng

Original citation: *J Clin Invest*. 2011;121(12):4670–4684. https://doi.org/10.1172/JCI58559

Citation for this retraction: *J Clin Invest*. 2025;135(18):e198611. https://doi.org/10.1172/JCI198611

Errors were recently identified in Figure 4, B, I, and K, and Supplemental Figure 5, J and K. Although the authors have indicated that original source data support the findings in these figure panels, the Editors are retracting the article due to the number of errors made.

Webster K. Cavenee agrees with the retraction. Shi-Yuan Cheng and Motoo Nagane dissent from the Journal’s decision to retract. The remaining authors abstained from commenting or could not be reached.

## Version 1 09/16/2025

Electronic publication

